# Clinical review of High Flow Nasal Cannula and Continuous Positive Airway Pressure in pediatric acute respiratory distress

**DOI:** 10.1016/j.amsu.2021.103180

**Published:** 2021-12-14

**Authors:** Kurniawan Taufiq Kadafi, Saptadi Yuliarto, Charity Monica, William Prayogo Susanto

**Affiliations:** Pediatric Emergency and Intensive Care Department, Saiful Anwar General Hospital, Brawijaya University, Malang, Indonesia

**Keywords:** Respiratory distress, HFNC, CPAP, Pediatric, Non-invasive ventilation, Clinical review

## Abstract

Acute Respiratory Distress Syndrome (ARDS) causes much morbidity and mortality in children. In mild to moderate ARDS, non-invasive ventilation (NIV) is the treatment of choice. Recently, there are 2 kinds of NIV used Continuous Positive Airway Pressure (CPAP) or High Flow Nasal Cannula (HFNC). Both of them can be used in various respiratory distress and have different physiological mechanisms. The effectiveness to improve the clinical parameter, morbidity, and mortality are similar between CPAP and HFNC. However, HFNC application is more tolerated in acute respiratory distress in children, with less nasal injury, lower heart rate inflicted, and better comfort index score.

## Highlights

• CPAP & HFNC widely used in pediatric acute respiratory distress.
• Both modalities have a different characteristics, beneficial in certain condition.
• The usage of each modalities depends on the causes of the respiratory distress.

## 1 Introduction

Acute Respiratory Distress Syndrome (ARDS) is one of the leading causes of morbidity and mortality in children. According to Berlin Criteria, ARDS on children can be evaluated from P/F ratio, or by Oxygenation Index (OI) and Oxygenation Saturation Index (OSI), according to Pediatric Acute Lung Injury Consensus Conference (PALICC). ARDS is classified into mild, moderate, and severe. ARDS incidence in children is 3.5 cases per 100.000 in the general population and 2.3 cases per 100.000 in children in PICU annually [[1], [2], [3]]– (see Table 1, Table 2)

**Table 1:** RCT Studies Described the Comparison of HFNC vs CPAP in Acute Respiratory Distress in Children.

Study	Method	Subject	Intervention	Outcome	Conclusion
1. Vitaliti G et al.*,* 2016	*Randomized controlled trial*	60 children, aged 1–24 months with mild-to-moderate respiratory distress (pneumonia, asthma, bronchiolitis)	CPAP helmet and HFNC application	Subject with CPAP helmet showed better improvement on blood pH (p = 0.043), PCO2 (p < 0.001), and P/F ratio (p < 0.001) after 1-h therapy, while subject with HFNC showed improvement in SpO2% (p = 0.009), PaO2 (p = 0.009), and P/F ratio (p = 0.009).	Both CPAP and HFNC proved to improve the clinical condition of the children with mild-to-moderate respiratory distress. However, CPAP helmets showed to induce clinical improvement faster than HFNC.
2. Milesi C et al., 2017	*Randomized Controlled Trial*	142 infants aged <6 months with moderate-to-severe acute bronchiolitis.	CPAP/HFNC application with the outflow of 2 L/Kg/minute.	Therapy failure occurred in 22/71 infants (31%) in the CPAP group and in 36/71 infants (50,7%) in the HFNC group. Risk analysis showed the 19% differences between the CPAP group and the HFNC group, but statistically not significant (95% CI -35 to −3%, p = 0,707). Intubation frequency, length of NIV, skin lesion, and length of PICU stay not significantly different (p > 0,05).	The first choice of treatment with HFNC had a higher level of failure than CPAP on young infants with moderate-to-severe acute bronchiolitis, however not statistically significant.
3.Ramnarayan P et al.*,* 2018	*Multi-centre Pilot Randomized Controlled Trial*	113 children with correction age >36 weeks until <16 years which fulfill the criteria of hypoxia, acute respiratory acidosis, or moderate respiratory distress.	HFNC/CPAP application	From 113 subjects (HFNC 59, CPAP 54), therapy switching from HFNC to CPAP (group A 44% and group B 21%) was more frequent than from CPAP to HFNC (group A 23% and group B 12%). First 72-h intubation more often in HFNC group than CPAP group (25,4% vs 18,5%, p = 0,38). The number of ventilator-free subjects in 28 days was lower in the HFNC group than in the CPAP group (group A: 19,6 vs 23,5 and group B: 21,8 vs 22,2).	Less number of subjects intubated in the first 72 h, number of therapy switching/escalation, length of PICU stay, number of ventilator-free subjects in the 28 days, and mortality rates in CPAP group than HFNC group. However, the differences are considered as not significant statistically (p > 0,05).
4.Sarkar M et al., 2018	*Randomized Controlled Trial (pilot study)*	31 infant aged 1–12 months with acute severe bronchiolitis.	CPAP or Hot Humidified High Flow Nasal Cannula Application	Subjective and functional parameters such as SpO2, RR, PaO2, PCO2, and RDAI were improved in both groups. HFNC group has a more significant HR decrease (p < 0.001), better COMFORT score (p < 0.003), and less nasal injury incidence (26,66% vs 75%, p = 0,021) than CPAP. Length of usage from CPAP (3,8 ± 0,8 days) and HFNC (3,6 ± 0,63 days) not significantly different (p = 0,33). Length of PICU stay between CPAP group (5 ± 1,788 days) and HFNC group (5 ± 1,6 days) not significantly different too (p = 0,105).	HFNC tolerated better than nCPAP in a patient with bronchiolitis.

**Table 2:** HFNC vs CPAP Outcome to Respiratory Distress in Children in several RCT Studies who Evaluate the Physiology, Morbidity, and Mortality Parameter.

Studies		Vitaliti G et al., 2016	Milesi C et al., 2017	Ramnarayan P et al., 2018	Sarkar M et al., 2018
**Physiologic parameter**	RR	RR decrease occurred faster in CPAP group, but statistically non-significant (p = 0,49 vs p=>0,99)	RR increase more frequently occurred in HFNC group (26,8% vs 11,3%, p = 0,03)		
	**PaCO2**	Significant decrease in CPAP group on 1 h after the initial therapy (p < 0,001 vs p = 0,9)			
	**mWCAS/RDAI score**		mWCAS score increase more frequently occured in HFNC group than CPAP group (29,6% vs 14,1%, p = 0,04)		RDAI score improvement not significantly different between both groups (p = 0,0967)
Morbidity	**Hospitalized duration**	Less hospitalization duration on both groups than the control group (p=<0,001). Hospitalization duration was longer in the HFNC group than the CPAP group (13,1 ± 1,32 vs 4,9 ± 1,1).	Length of PICU stay not significantly different between both groups (p = 0,44)		Length of PICU stay not significantly different between both groups (p = 0,105)
	**Pain/NIPS/COMFORT/EDIN score**		EDIN score improvement more frequently occrued in CPAP group (18,3% vs 8,5%, p = 0,14)	Higher mean modified COMFORT score in patients who not tolerate CPAP than HFNC in the first 6-h of therapy (19 SD 4,4 vs 15,3 SD 3,1)	COMFORT score decrease more significantly in the HFNC group (p < 0,003)
	**Therapy Escalation Frequency**		Intubation frequency was not significantly different between both groups (p = 0,72).	Intubation frequency in the first 72 h was higher in the HFNC group, but statistically not significant (25,4% vs 18,5%, p = 0,38)	
	**Therapy duration**		Therapy duration not significantly different between both groups (p = 0,225)		Therapy duration not significantly different between both groups (p = 0,33)
	**Nasal injury**		Nasal injury incidence not significantly different between both groups (p = 0,27)		Significant less nasal injury incidence on HFNC group (p = 0,021)
Mortality			All of the patients survived	Higher PICU mortality in HFNC group (5,1% vs 3,7%).	

Non-Invasive Ventilation (NIV) recently become the treatment of choice in children with mild to moderate ARDS and a P/F ratio of more than 150. Several diseases can be treated by NIV, such as restrictive lung disease (on the neuromuscular disorder), obstructive sleep apnea (e.g in children with Down Syndrome), pneumonia, lower respiratory tract obstruction (e.g asthma and bronchiolitis), and other causes of ARDS [4,5]. One of the NIV methods commonly used in PICU is Continuous Positive Airway Pressure (CPAP). CPAP application has a weakness, which requires additional tools, such as a mask or nasal prong, to prevent the air leak from the ventilator circuit. These tools cause any discomfort or even injury with prolonged use for the patient, which potentially induces treatment failure. CPAP also has side effects of inducing complications, such as pneumothorax or pneumomediastinum [5,6].

Another NIV modality for ARDS in children is High Flow Nasal Cannula (HFNC). HFNC delivers a high flow of humidified air into the children's respiratory tract. This high flow aims to decrease airway resistance, reduce dead space, and provide positive pressure to the airway [6]. This review provides an overview of the available option of NIV treatment in children with ARDS with more focus on the comparison between HFNC and CPAP. In this study, we summarize several studies which described the modalities used as an NIV treatment in children with ARDS. We provides the physiology and mechanism of the HFNC and CPAP, then we showed the comparison of both NIV methods in pediatric ARDS.

## 2 Pediatric Acute Respiratory Distress Syndrome (ARDS)

Historically, ARDS characteristics in children were made based on the adult criteria by American-European Consensus Conference (AECC) in 1994, then by Berlin criteria in 2012. However, it was known that ARDS in children had different characteristics from adults and new definitions and guidelines were made by Pediatric Acute Lung Injury Consensus Conference (PALICC). Pediatric ARDS (PARDS) classification was simplified by PALICC to accommodate the requirement in healthcare facilities with limited resources, in which the blood gas analysis cannot be done immediately [[7], [8], [9]].

Classification of mild, moderate, or severe PARDS can be made based on oxygen saturation. PALICC criteria using Oxygenation Index (OI) (calculation: FiO_2_ x mean airway pressure x 100/PaO_2_) to classify the PARDS, not the P/F ratio, with invasive mechanical ventilation. Using the oxygen saturation, PALICC provides Oxygen Saturation Index (OSI) (calculation: FiO_2_ x mean airway pressure x 100/SpO_2_) if the OI cannot be calculated. However, in children with NIV with CPAP minimal pressure of 5 cmH_2_O, it is recommended to use the P/F ratio to classify the ARDS severity. Patients with NIV in the limited resources health facility can use the OSI to determine the ARDS severity. These criteria can be used with titration of the mechanical ventilation to achieve oxygen saturation of 88–97% [[7], [8], [9]].

## 3 High Flow Nasal Cannula (HFNC)

### 3.1 HFNC physiology & mechanism

HFNC is one of the NIV modalities other than CPAP. Minimum oxygen flow to be classified as high flow is different in various literature. One of them mentions minimum oxygen flow of 2L/minute on the neonate and 4–6 L/min on children can be classified as high flow. The important feature of HFNC application is the mechanism to warm and humidify the air before high flow is administered. Naturally, oxygen is dry and the bubble humidifier which is commonly used in nasal cannula cannot adequately humidify the air on 3–5L/minute flow [10].

Basic components of HFNC are a pressurized oxygen source, which is regulated by a flowmeter and blender, a sterile air reservoir that attaches to the heater and humidifier, a close circuit with a warmer to maintain the temperature and humidity, and a non-occlusive nasal cannula interface. There are several mechanisms of HFNC to reduce the PARDS, as followed.

1. Reduce the breathing effort. High flow oxygen decreases the airway resistance on inspiration, thus can reduce the breathing effort.
2. Reduce energy expenditure. Oxygen with adequate humidity reduces the evaporation in upper airway mucosa, decrease the energy expenditure for the metabolism.
3. Improve lung compliance and mucociliary function. Dry and cold airflow can induce bronchoconstriction, thus warming and humidifying the airflow will reduce it.
4. Wash out the nasopharyngeal dead space. On HFNC application, the nasal cavity and oropharynx continuously washed and flowed with oxygen. This condition reduces rebreathing, improves the exhalation airflow, and increases the carbon dioxide outflow. HFNC requires an open system to work, thus the nasal cannula must not cover more than half of the nostril diameter when applicated and the mouth should constantly open.
5. Give positive pressure to the airway. The positive pressure gained from this device depends on the oxygen flow, patient body weight, and relative proportion of nasal cannula to the nostril. Pressure increases with higher oxygen airflow but decreases with elder age and bigger body weight [10].

### 3.2 HFNC treatment on children with respiratory distress

Several studies showed positive pressure on the airway obtained from HFNC can open the collapsed alveoli and increase the lung functional residual capacity (FRC). HFNC is frequently used as therapy in PARDS, as HFNC provides more effective treatment and is quite comfortable for children [11]. Kepreotes et al. reported HFNC (1L/kg/minute until 20L/minute flow) less likely to develop treatment failure compared to standard oxygenation with low flow nasal cannula (100% oxygen in 2L/minute flow) in children with moderate bronchiolitis. Only 14% of children with HFNC have treatment failure, compared with 335 from low flow nasal cannula (p = 0.0016) [12]. A meta-analysis and systematic review from Lin et al. analyze 9 randomized controlled trials (RCT) which compares HFNC with standard oxygen therapy. HFNC showed to have significantly less therapy failure than standard oxygen therapy (RR 0.5; CI 0.4–0.62; p < 0.01) [13].

The oxygen outflow of HFNC to get the optimal effect was already reported from several studies and various results were gained. Children below 2 years of age or with less than 10 kg of body weight, reported to have a benefit with HFNC 1–2 L/kg/minute and could be increased by 0.5 L/kg/minute. However, an outflow of 3 L/kg/minute causes discomfort to the patient [11,14]. A Study from Milesi et al. compared 142 babies with HFNC 2 L/kg/minute and 144 babies with HFNC 3 L/kg/minute. They showed no significant differences in treatment failure between the two treatments and worsening respiratory distress was the main reason for treatment failure [14].

## 4 Continuous Positive Airway Pressure (CPAP)

### 4.1 CPAP physiology & mechanism

CPAP is a device to give positive pressure to the airway to maintain the patent airway. However, CPAP can only be applied in a patient with spontaneous breath. CPAP not only provides Positive End-Expiratory Pressure/PEEP (alveolus pressure in the expiratory end higher than atmospheric pressure) but also maintains pressure along the respiratory tract, in inspiration and expiration [15]. CPAP works on several mechanisms, as followed.

• Reduce upper airway tract resistance and overcome the obstruction
• Increase tone and contractility of the diaphragm muscles
• Improve lung compliance
• Increase lung tidal volume with low FRC
• Improve ventilation/perfusion ratio and reduce oxygen demand
• Maintain surfactant on alveoli surface and reduce the alveoli edema

All of these mechanisms had a role to maintain lung FRC and improve oxygenation [16].

CPAP works on 2 methods, variable flow and continuous flow. CPAP with ventilator machine (conventional CPAP) and bubble CPAP are types of continuous flow. Infant Flow Driver and Benveniste gas-jet valve CPAP are the types of variable flow. Variable flow CPAP builds the pressure in the proximal airway tract of nares, using the Bernoulli Effect to change the airflow and maintain the pressure [[17], [18], [19]].

### 4.2 CPAP treatment on children with respiratory distress

Several studies showed the benefit of CPAP application in PARDS. Jayashree et al. compared the usage of bubble CPAP and nasal prong in children aged 1 month to 12 years with respiratory distress. They reported more clinical deterioration in patients with a nasal prong (25.9%) than bubble CPAP (1.8%) [20]. Another study from Wilson et al. compared the usage of CPAP with control in children aged 1 month to 5 years with respiratory distress for 2 weeks. They reported 3% (26 from 1021) mortality rates in CPAP patients and 4% (44 from 1160) mortality rates in control patients. There was a lower mortality risk in CPAP patients, with a relative risk (RR) of 0.67 (95%CI 0.42–1.08, p = 0.11). In this same study for children <1 year old, the differences in mortality rates were significant, with 3% (10 from 374) in CPAP patients and 7% (24 from 359) in control patients (RR 0.4; 95%CI 0.19–0.82, p = 0.01) [21].

## 5 Comparison between HFNC & CPAP on children with acute respiratory distress

Numerous studies show the comparison between HFNC and CPAP application in children with respiratory distress. Vitaliti et al. tested the effectivity of HFNC and CPAP as a therapy in 60 children with mild-to-moderate respiratory distress, divided into 3 groups, HFNC, CPAP, and control, each consisting of 20 patients. Bronchiolitis dominated the causation of respiratory distress with 31 patients (51.67%). 6 parameters were evaluated, including SpO_2_, PaO_2_, PaCO_2_, pH, respiratory rate, and P/F ratio, in 3 periods: onset of therapy, 1-h of therapy, and 6-h of therapy. All of the parameters were improving within 6 h of therapy with HFNC and CPAP. However, the significant improvement with HFNC obtained from SpO_2_ (p = 0.009), PaO_2_ (p = 0.009), and P/F ratio (p = 0.009), while with CPAP, the significant improvement gained from pH (p = 0.043), PaCO_2_ (p=<0.001), and P/F ratio (p=<0.001) [22].

Studies from Sarkar et al. compared the effectivity of HFNC and CPAP in severe bronchiolitis children with SpO2 <92% and Respiratory Distress Assessment Index (RDAI) score ≥11. 16 patients received CPAP with a nasal mask or nasal prong interface, starting from 4 cm H_2_O and gradually raising to 8 cm H_2_O. Meanwhile, 15 patients received HFNC with 2 L/kg/minute flow for children ≤10 kg and the addition of 0.5 L/kg/minute for children >10 kg. Oxygen fraction starts on 0.4, then gradually increased to reach the SpO_2_ target of 94%. Clinical parameters showed similar improvement in both treatments, only 1 patient of each group deteriorated and required intubation (p = 0.29). Length of stay in PICU was also similar between both treatments (p = 0.105). However, HFNC seems to be more tolerated with a lower heart rate (p < 0.001), better comfort score index (p = 0.003), and lower nasal injury (p = 0.021) in children with severe bronchiolitis [23].

Opposite results showed by Pedersen et al. who reported CPAP application more effectively to reduce RR and FiO2 than HFNC in children with bronchiolitis. More than half of the HFNC patients deteriorated and switched to CPAP. Length of PICU stay and hospitalization is similar between both groups. However, there was a weakness in this study, since it was a retrospective study and no randomization was performed [24]. Another RCT from Liu et al. compared CPAP and HFNC in children less than 2 years old with mild-to-moderate respiratory distress caused by pneumonia. They reported no significant differences in treatment effectivity, length of stay, and clinical deterioration between HFNC and CPAP application. Nasal mucose injury and abdominal distention were more frequently found in CPAP [25].

Studies from Ramnarayan et al. aimed to compare the effectiveness of HFNC and CPAP to children <16 years old with respiratory distress in different conditions. The NIV was performed on two groups of patients, one group with acute respiratory disease who require immediate NIV intervention (step-up) and one group with deterioration after extubation who also require NIV application (step-down). Both HFNC and CPAP showed similar improvement in the clinical outcome, also had non-significant differences in re-intubation frequency, length of PICU stay, and mortality rates [6].

Other studies of respiratory distress in children reported by Milesi et al. who compared HFNC and CPAP in children <6 months with acute bronchiolitis and severe respiratory distress, classified from mWCAS (modified Wood's Clinical Asthma Score) >3. In this study, CPAP had a better effectivity with less clinical deterioration, 22 from 71 (31%) in CPAP and 36 from 71 (50.7%) in HFNC (RR 1.63; 95%CI 1.02–2.63, p = 0.001). The onset of treatment failure was similar in both NIV (6.7 h in CPAP and 9.7 h in HFNC, p = 0.19). The causation of treatment failure is mWCAS score increase (31 cases), RR increase (27 cases), EDIN score increase (19 cases), and apnea (7 cases). The main factor of therapy failure in CPAP was the discomfort and in HFNC was the worsening of respiratory distress. Based on univariate analysis, the therapy failure predictor in CPAP was higher body weight (p = 0.04), while in the HFNC group was higher initial FiO_2_ (p = 0.02). 8 subjects require therapy escalation into intubation, which tends to correlate with HFNC therapy failure (p = 0.054). HFNC therapy failure mainly occurred in the first 6-h with worsening of respiratory distress, which 2/3 of them can be improved with CPAP [14].

## 6 Conclusion

HFNC and CPAP as an NIV provides a similar improvement in clinical parameter and reduce morbidity and mortality in children with acute respiratory distress. CPAP is more efficient to reduce the respiratory muscle requirement in respiratory distress. HFNC is more tolerated than CPAP with less nasal mucose injury, lower heart rate, and better comfort index score. NIV application on children with respiratory distress must be determined based on several factors, such as the causation of disease, severity of the distress, and body weight. There is a limitation in this study, considering we summarize several studies which showed the comparison of HFNC and CPAP as NIV in children and did not statistically analyze the comparison.

## Provenance and peer review

Not commissioned, externally peer reviewed.

## Ethical approval

This is a review journal, thus the ethical approval is irrelevant with this study.

## Source of funding

This study was funded by Pediatric Department of Medical Faculty, Brawijaya University (ID: 171/UN10.F08.16.42/KP/2021)

## Author contribution

1. Kurniawan Taufiq Kadafi: design the study concept, collect & summarize the associated references, review the manuscript
2. Saptadi Yuliarto: design the study concept, collect & summarize the associated references, review the manuscript
3. Charity Monica: collect & summarize the associated references, writing the paper
4. William Prayogo Susanto: summarize the references, writing the paper

## Trial registry number

This is a review study, thus no human trials performed and registered.

## Guarantor

The guarantor of this study is Kurniawan Taufiq Kadafi as the corresponding author.

## Consent

This is a review study, thus informed consent form is irrelevant with this study.

## Declaration of competing interest

None of the authors have a conflict of interest.
